# Supplementary material for: The relative effects of self-reported noise and odour annoyance on psychological distress: Different effects across sociodemographic groups?
Source: PLoS One. 2021 Oct 1;16(10):e0258102. doi: 10.1371/journal.pone.0258102 (PMC8486134; doi:10.1371/journal.pone.0258102)
Supplement: S1 Appendix — (DOCX) [file pone.0258102.s001.docx]

**S1 Appendix**

**Table A1. Average psychological distress for different levels of noise and odour annoyance (adult sample)**

|  | Noise annoyance | Odour annoyance - agriculture | Odour annoyance - other |
| --- | --- | --- | --- |
| Not annoyed (score from 0 to under 3) | 14.9 | 15.3 | 15.3 |
| Somewhat annoyed (score from 3 to under 8) | 21.1 | 18.9 | 22.5 |
| Highly annoyed (8-10) | 31.5 | 21.3 | 20.2 |

Source: Health Monitor (2016).


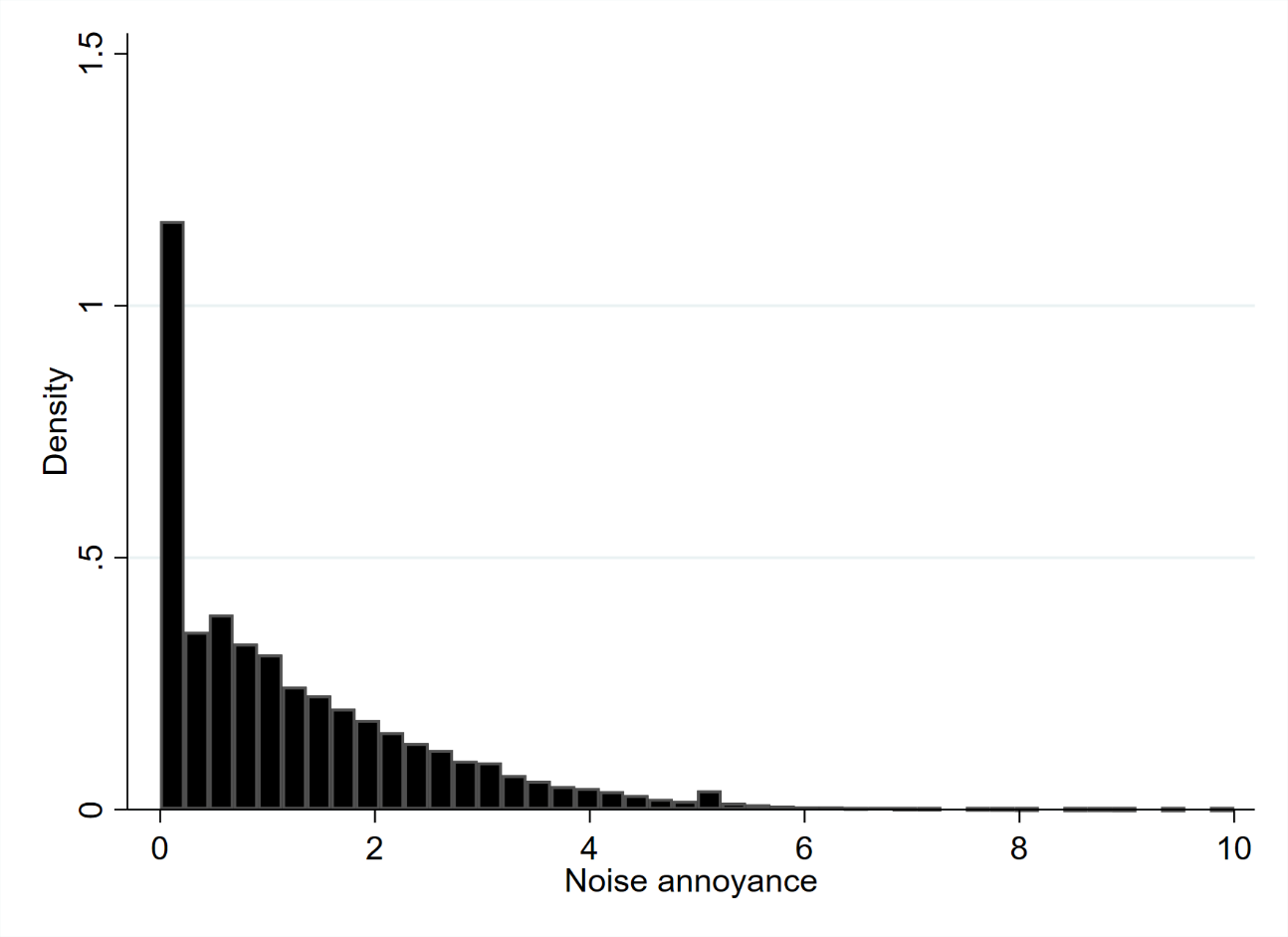


**Fig 1. Histogram of division of scores on noise annoyance**


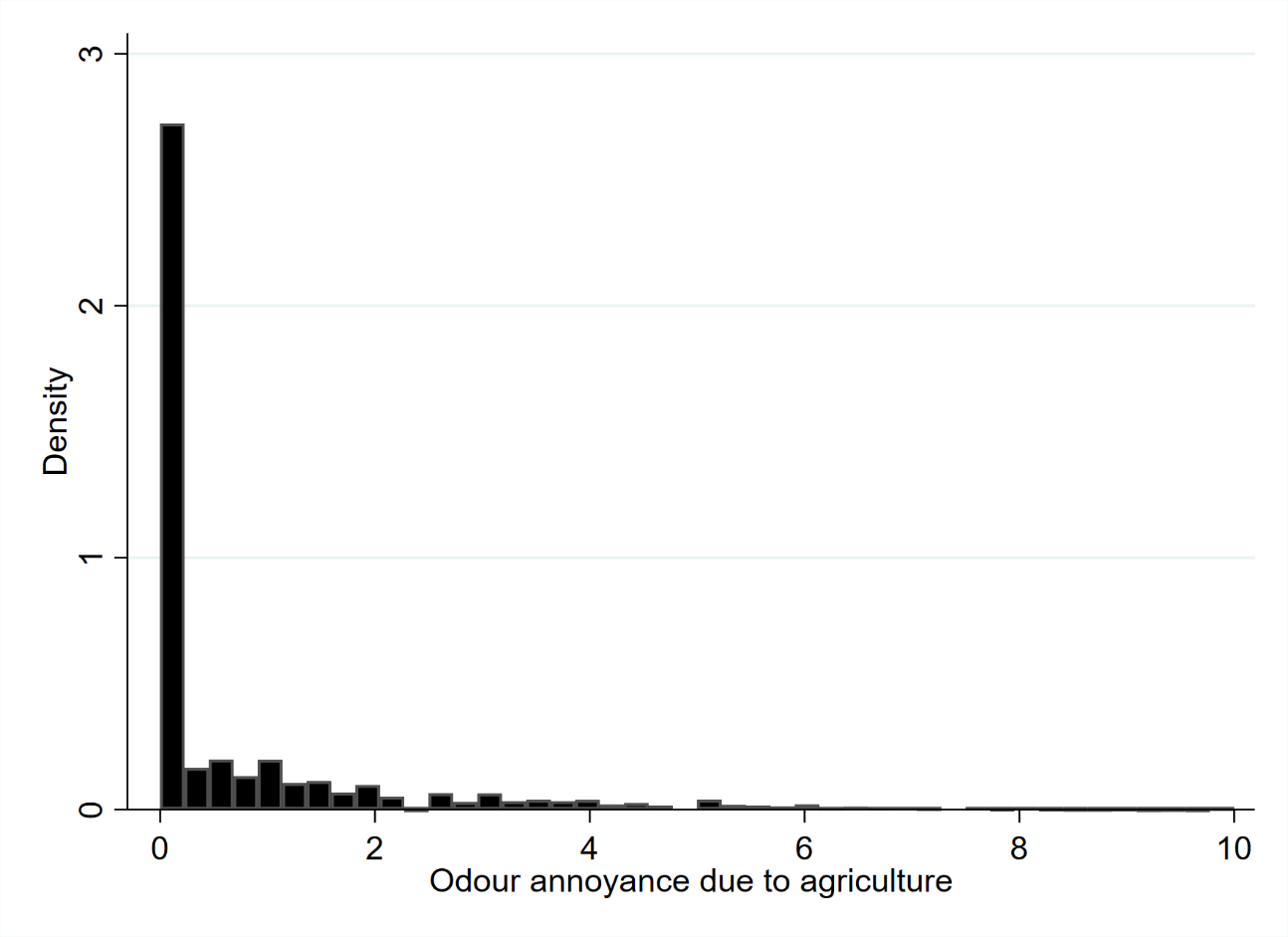


**Fig 2. Histogram of division of scores on odour annoyance due to agriculture**


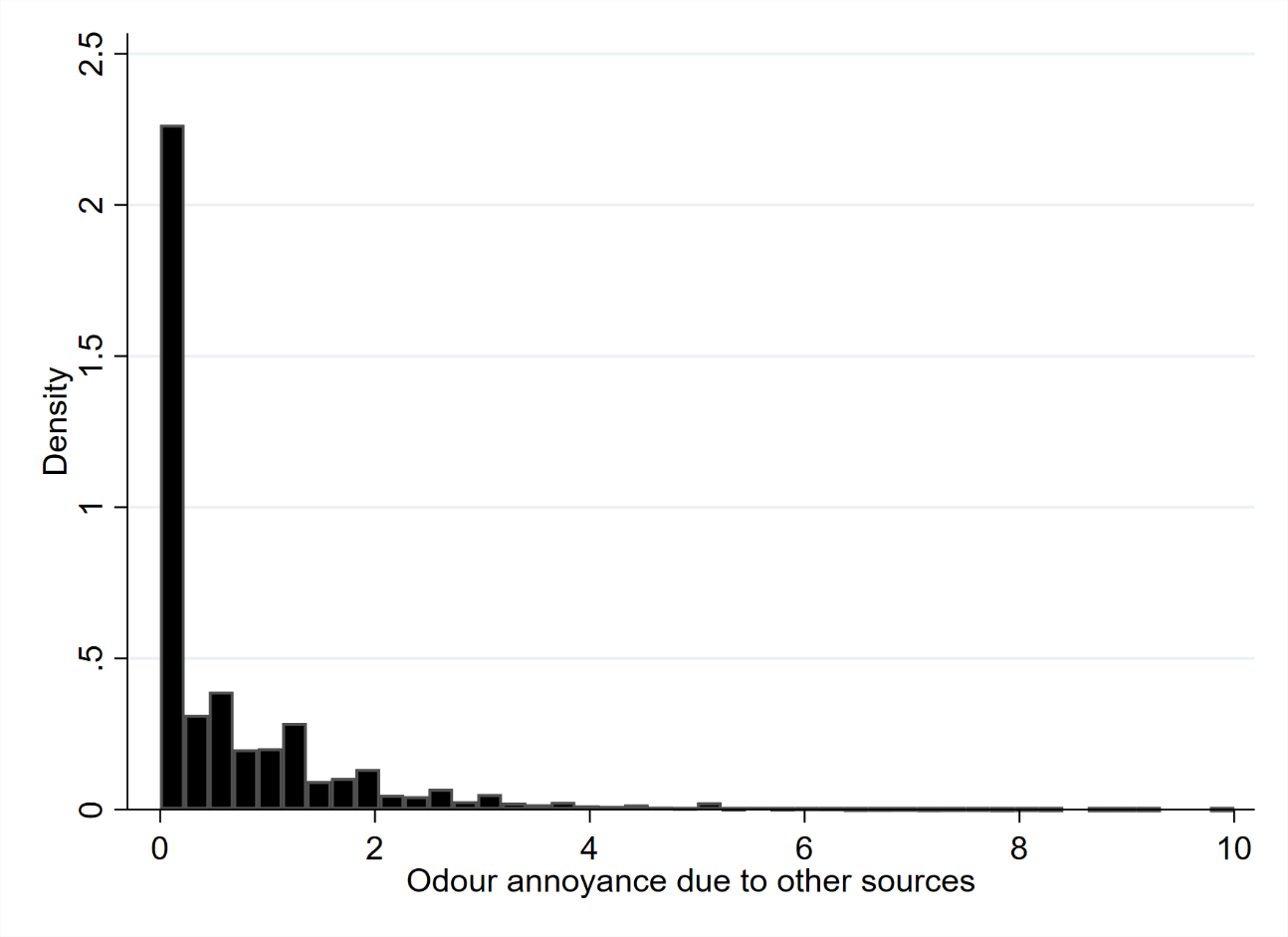


**Fig 3. Histogram of division of scores on odour annoyance due to other sources**

**Table A2. Descriptive statistics of all variables for the sample with adults and older adults (n = 34838)**

|  | Min | Max | Mean | | SD | | |  |
| --- | --- | --- | --- | --- | --- | --- | --- | --- |
| Psychological distress | 0 | 100 | 15.21 | | 15.41 | | |  |
| Noise annoyance | 0 | 10 | 0.70 | | 1.28 | | |  |
| Odour annoyance – agriculture | 0 | 10 | 0.81 | | 1.61 | | |  |
| Odour annoyance – other | 0 | 10 | 0.66 | | 1.05 | | |  |
| *Age category* |  |  |  | |  | | |  |
| Young adults |  |  | 15.3% | |  | | |  |
| Young middle-aged |  |  | 22.0% | |  | | |  |
| Older middle-aged |  |  | 35.4% | |  | | |  |
| Older adult |  |  | 27.3% | |  | | |  |
| Female |  |  | 52.6% | |  | | |  |
| *Educational level* |  |  |  | |  | | |  |
| No / primary |  |  | 7.5% |  | |  |  |  |
| Lower secondary |  |  | 36.0% |  | |  |  |  |
| Higher secondary |  |  | 28.9% |  | |  |  |  |
| Tertiary |  |  | 27.6% |  | |  |  |  |
| *Control variables* |  |  |  |  | |  |  |  |
| Lives with children under 18 |  |  | 22.4% |  | |  |  |  |
| Self-rated health |  |  |  | |  | | |  |
| (Very) Good |  |  | 76.0% | |  | | |  |
| Moderate |  |  | 20.4% | |  | | |  |
| (Very) Bad |  |  | 3.6% | |  | | |  |
| *Subjective financial wellbeing* |  |  |  | |  | | |  |
| Yes vs. no |  |  | 12.8% | |  | | |  |
| *Self-reported risks in the environment* |  |  |  | |  | | |  |
| Lives in a busy street |  |  | 23.1% | |  | | |  |
| Lives near industry |  |  | 15.3% | |  | | |  |
| Living near airport |  |  | 12.7% | |  | | |  |
| Living near a livestock farm |  |  | 23.5% | |  | | |  |
| Living near a wind turbine |  |  | 2.3% | |  | | |  |
| Living near agriculture (without livestock) |  |  | 28.3% | |  | | |  |
| Living near a route for dangerous materials |  |  | 8.5% | |  | | |  |
| Living near a gas station |  |  | 15.5% | |  | | |  |

Source: Health Monitor (2016).

**Table A3. Selected effects of OLS regression for psychological distress (n = 34838)**

|  | Model 1 |  |  | Model 2 |  |  | Model 3 |  |  |
| --- | --- | --- | --- | --- | --- | --- | --- | --- | --- |
|  | B (se) | Beta | P-value | B (se) | Beta | P-value | B (se) | Beta | P-value |
| Noise annoyance | 1.41 (0.06) | 0.12 | 0.00 | - | - | - | 1.00 (0.07) | 0.08 | 0.00 |
| Odour annoyance – agriculture | - | - | - | 0.31 (0.06) | 0.03 | 0.00 | 0.21 (0.06) | 0.02 | 0.00 |
| Odour annoyance – other | - | - | - | 1.40 (0.09) | 0.10 | 0.00 | 0.92 (0.10) | 0.06 | 0.00 |
| *Age category (ref. young adults)* |  |  |  |  |  |  |  |  |  |
| Young middle-aged | -2.89 (0.26) | -0.08 | 0.00 | -3.11 (0.26) | -0.08 | 0.00 | -3.05 (0.26) | -0.08 | 0.00 |
| Older middle-aged | -4.13 (0.25) | -0.13 | 0.00 | -4.50 (0.25) | -0.14 | 0.00 | -4.41 (0.25) | -.0.14 | 0.00 |
| Older adults | -4.73 (0.30) | -0.14 | 0.00 | -5.21 (0.30) | -0.15 | 0.00 | -4.99 (0.30) | -0.14 | 0.00 |
| Female | 2.79 (0.16) | 0.09 | 0.00 | 2.85 (0.16) | 0.09 | 0.00 | 2.81 (0.16) | 0.09 | 0.00 |
| *Educational level (ref. tertiary)* |  |  |  |  |  |  |  |  |  |
| No / primary | 9.29 (0.43) | 0.12 | 0.00 | 9.08 (0.43) | 0.11 | 0.00 | 9.25 (0.43) | 0.12 | 0.00 |
| Lower secondary | 3.57 (0.22) | 0.10 | 0.00 | 3.31 (0.22) | 0.10 | 0.00 | 3.52 (0.22) | 0.10 | 0.00 |
| Higher secondary | 1.42 (0.19) | 0.04 | 0.00 | 1.27 (0.19) | 0.04 | 0.00 | 1.38 (0.19) | 0.04 | 0.00 |
| *Control variables* |  |  |  |  |  |  |  |  |  |
| Parenthood | - | - | - | - | - | - | - | - | - |
| Self-rated health (ref. good) |  |  |  |  |  |  |  |  |  |
| Moderate | - | - | - | - | - | - | - | - | - |
| Bad | - | - | - | - | - | - | - | - | - |
| *Lives near:* |  |  |  |  |  |  |  |  |  |
| A busy street | 0.41 (0.19) | 0.01 | 0.03 | 0.40 (0.19) | 0.01 | 0.04 | 0.22 (0.19) | 0.01 | 0.25 |
| Industry | -0.61 (0.24) | -0.01 | 0.01 | -0.27 (0.24) | -0.01 | 0.25 | -0.65 (0.24) | -0.02 | 0.01 |
| Airport | 0.30 (0.31) | 0.01 | 0.34 | 0.03 (0.32) | 0.00 | 0.93 | 0.03 (0.31) | 0.00 | 0.94 |
| Livestock farm | 0.46 (0.26) | 0.01 | 0.08 | 0.02 (0.27) | 0.00 | 0.96 | 0.14 (0.27) | 0.00 | 0.60 |
| Wind turbine | 0.64 (0.58) | 0.01 | 0.27 | 0.67 (0.59) | 0.01 | 0.26 | 0.62 (0.58) | 0.01 | 0.29 |
| Agriculture (without livestock) | -0.08 (0.25) | -0.00 | 0.76 | -0.22 (0.25) | -0.01 | 0.38 | -0.14 (0.25) | 0.01 | 0.56 |
| A route for dangerous materials | 0.52 (0.30) | 0.01 | 0.08 | 0.27 (0.30) | 0.01 | 0.36 | 0.24 (0.30) | 0.00 | 0.41 |
| Gas station | 0.13 (0.23) | 0.00 | 0.58 | 0.15 (0.23) | 0.00 | 0.52 | 0.10 (0.23) | 0.00 | 0.66 |
| Intercept | 9.81 (1.88) |  | 0.00 | 10.38 (1.88) |  | 0.00 | 9.91 (1.87) |  | 0.00 |
| *Explained variance* | 13.3 |  |  | 13.2 |  |  | 13.7 |  |  |

Notes: all models are controlled for subjective financial wellbeing and indicator variables for all districts (ref. Wijk en Aalburg).

Source: Health Monitor (2016).

**Table A4. Selected effects of OLS regression for psychological distress (n = 34838)**

|  | Model 1 |  |  | Model 2 |  |  | Model 3 |  |  |
| --- | --- | --- | --- | --- | --- | --- | --- | --- | --- |
|  | B (se) | Beta | P-value | B (se) | Beta | P-value | B (se) | Beta | P-value |
| Noise annoyance | 0.98 (0.07) | 0.08 | 0.00 | 0.84 (0.06) | 0.07 | 0.00 | 0.83 (0.06) | 0.07 | 0.00 |
| Odour annoyance – agriculture | 0.20 (0.06) | 0.02 | 0.00 | 0.21 (0.06) | 0.02 | 0.00 | 0.21 (0.06) | 0.02 | 0.00 |
| Odour annoyance – other | 0.93 (0.10) | 0.06 | 0.00 | 0.73 (0.09) | 0.05 | 0.00 | 0.74 (0.09) | 0.05 | 0.00 |
| *Age category (ref. young adults)* |  |  |  |  |  |  |  |  |  |
| Young middle-aged | -2.13 (0.27) | -0.06 | 0.00 | -3.55 (0.24) | -0.10 | 0.00 | -2.93 (0.25) | -0.08 | 0.00 |
| Older middle-aged | -4.91 (0.25) | -0.15 | 0.00 | -5.86 (0.22) | -0.18 | 0.00 | -6.18 (0.23) | -.0.19 | 0.00 |
| Older adults | -5.66 (0.30) | -0.16 | 0.00 | -7.25 (0.27) | -0.21 | 0.00 | -7.68 (0.27) | -0.22 | 0.00 |
| Female | 2.84 (0.16) | 0.09 | 0.00 | 2.76 (0.14) | 0.09 | 0.00 | 2.77 (0.14) | 0.09 | 0.00 |
| *Educational level (ref. tertiary)* |  |  |  |  |  |  |  |  |  |
| No / primary | 9.08 (0.43) | 0.11 | 0.00 | 4.86 (0.39) | 0.06 | 0.00 | 4.76 (0.39) | 0.06 | 0.00 |
| Lower secondary | 3.32 (0.22) | 0.10 | 0.00 | 1.99 (0.20) | 0.06 | 0.00 | 1.87 (0.20) | 0.05 | 0.00 |
| Higher secondary | 1.26 (0.19) | 0.04 | 0.00 | 0.75 (0.17) | 0.02 | 0.00 | 0.68 (0.17) | 0.02 | 0.00 |
| *Control variables* |  |  |  |  |  |  |  |  |  |
| Parenthood | -2.51 (0.24) | -0.07 | 0.00 | - | - | - | -1.67 (0.21) | -0.05 | 0.00 |
| Self-rated health (ref. good) |  |  |  |  |  |  |  |  |  |
| Moderate | - | - | - | 11.97 (0.18) | 0.31 | 0.00 | 11.92 (0.18) | 0.31 | 0.00 |
| Bad | - | - | - | 25.62 (0.39) | 0.31 | 0.00 | 25.53 (0.39) | 0.31 | 0.00 |
| *Lives near:* |  |  |  |  |  |  |  |  |  |
| A busy street | 0.22 (0.19) | 0.01 | 0.25 | 0.14 (0.17) | 0.00 | 0.43 | 0.14 (0.17) | 0.00 | 0.42 |
| Industry | -0.66 (0.24) | -0.02 | 0.01 | -0.44 (0.21) | -0.01 | 0.04 | -0.44 (0.21) | -0.01 | 0.04 |
| Airport | 0.02 (0.31) | 0.00 | 0.96 | 0.10 (0.28) | 0.00 | 0.73 | 0.09 (0.28) | 0.00 | 0.74 |
| Livestock farm | 0.15 (0.27) | 0.00 | 0.58 | 0.08 (0.24) | 0.00 | 0.75 | 0.08 (0.24) | 0.00 | 0.74 |
| Wind turbine | 0.64 (0.58) | 0.01 | 0.28 | 0.56 (0.53) | 0.01 | 0.29 | 0.56 (0.53) | 0.01 | 0.29 |
| Agriculture (without livestock) | -0.11 (0.24) | -0.00 | 0.64 | 0.06 (0.22) | 0.00 | 0.79 | 0.08 (0.22) | 0.00 | 0.73 |
| A route for dangerous materials | 0.24 (0.30) | 0.00 | 0.43 | 0.26 (0.27) | 0.00 | 0.33 | 0.26 (0.27) | 0.00 | 0.33 |
| Gas station | 0.04 (0.23) | 0.00 | 0.86 | -0.03 (0.21) | -0.00 | 0.89 | -0.07 (0.21) | -0.00 | 0.74 |
| Intercept | 10.91 (1.87) |  | 0.00 | 9.40 (1.69) |  | 0.00 | 10.07 (1.69) |  | 0.00 |
| *Explained variance* | 13.9 |  |  | 29.4 |  |  | 29.5 |  |  |

Notes: all models are controlled for subjective financial wellbeing and indicator variables for all districts (ref. Wijk en Aalburg).

Source: Health Monitor (2016).

**Table A5. Interaction effects of noise annoyance for different age groups**

|  | B (SE) | Beta | P-value |
| --- | --- | --- | --- |
| Noise annoyance | 1.19 (0.14) | 0.10 | 0.00 |
| *Interaction with (ref. young adults)* |  |  |  |
| Young middle-aged | -0.28 (0.18) | -0.01 | 0.13 |
| Older middle-aged | -0.42 (0.16) | -0.02 | 0.01 |
| Older adults | -0.57 (0.18) | -0.01 | 0.00 |

Notes: all models are controlled for subjective financial wellbeing, educational level, self-rated health, self-reported risks in the environment and indicator variables for all districts (ref. Wijk en Aalburg).

Source: Health Monitor (2016).


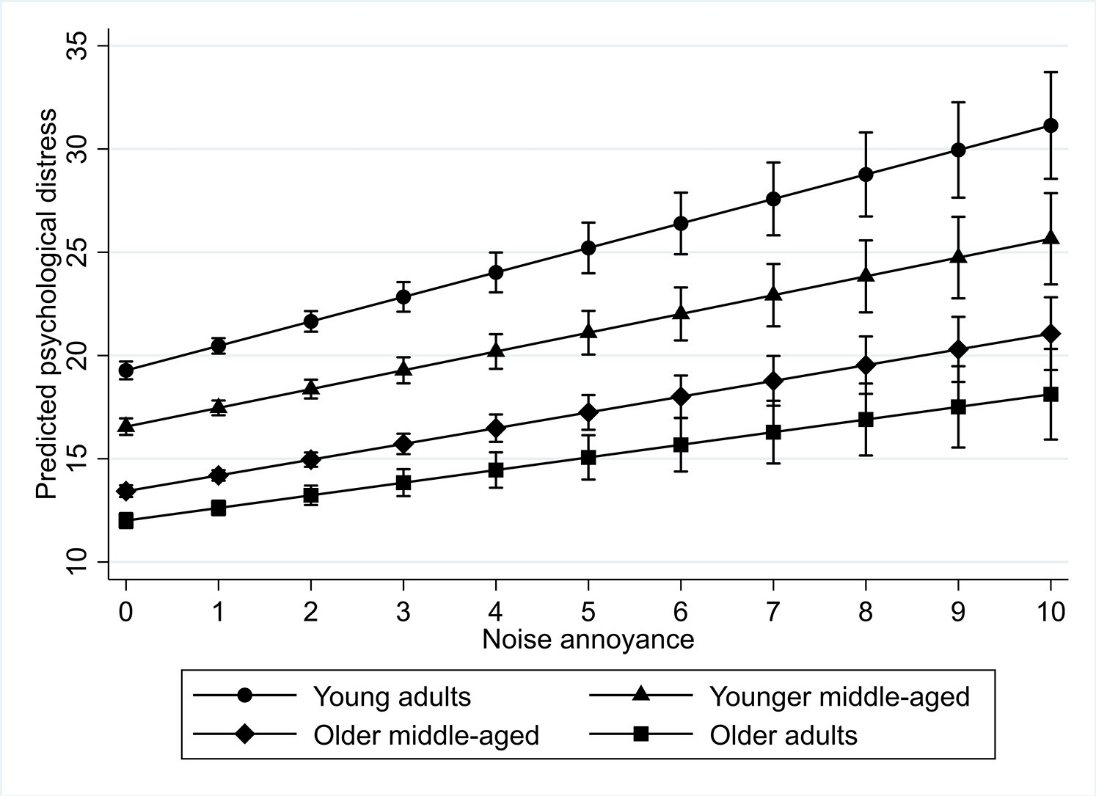


**Fig 4. Interaction plot for predicted psychological distress among different age groups by level of subjective noise annoyance**
