# Supplementary material for: The relative effects of self-reported noise and odour annoyance on psychological distress: Different effects across sociodemographic groups?
Source: PLoS One. 2021 Oct 1;16(10):e0258102. doi: 10.1371/journal.pone.0258102 (PMC8486134; doi:10.1371/journal.pone.0258102)
Supplement: Attachment — Submitted filename: Final revision letter.docx [file pone.0258102.s003.docx]

**Revision letter:**

***Comments by editor:***

1. ***Journal requirements***

We have carefully checked and adjusted our references (using the Plos One Endnote template) and figures (using the recommended site) to meet the journal requirements.
In addition, we are willing to make use of a proofreading service to check and improve the quality of writing if the editor requires us to do so.

2. ***Additional Editor Comments (if provided):*

   The reviewer comments were correctly addressed, and a number of final, minor revisions were requested. It is indeed important to address the reviewer comments by making changes to the manuscript**, **instead of just replying in the rebuttal letter. Readers of the manuscript might have the same questions as the reviewers, but they will of course not have access to the rebuttal letter.**

We are very grateful for all the useful feedback we have received in the previous revision. We are happy to note that the reviewers agree that we have handled the initial comments well and that the manuscript has improved greatly.

In this new version of the paper, we have addressed the reviewer comments and reviewed our revision letter to check if we have left out crucial information for the readers.

**Reviewer #2:**

**The manuscript greatly improved over the previous version, and, most importantly, there was quite a lot of additional important information (that was previously lacking) in the author's reply. For the manuscript to be acceptable, I suggest a minor revision which includes a few more of the thoughts from the detailed and well written reply letter, namely**

(1) **on the research rationale,**

We have now added information from the previous revision letter in the text. This includes information on why noise and odour annoyance is important from a policy perspective:

Moreover, noise and odour form the top two environmental complaints for European citizens and therefore is an important issue for policy (D-noses consortium, 2019). For policy makers at a municipality or province, sophisticated exposure data is generally hard to interpret and translate into policy. Therefore, studying the relationship between noise and odour annoyance and psychological distress is important, both from a scientific and policy perspective. (page 4)

Moreover, we have added more information about the impact of daily hassles such as noise and odour annoyance and how they contribute to health inequalities across different groups on page 5:

Life is full of low-impact factors (daily hassles, like noise and odour annoyance) that add up and use the coping resources of individuals and in this way have a substantial impact on individuals (Lazarus & Folkman, 1984). Different sociodemographic groups have access to different coping resources and have different daily living patterns, which influence their capacity to cope with environmental stressors. As a result, certain groups might experience higher levels of psychological distress due to perceived noise and odour annoyance. Answering the question whether different groups are impacted differently by these stressors therefore helps to understand the causes of health inequalities across different groups.

(2) **on the importance of odour and noise annoyance's contribution to general health,**

We have discussed this point in the beginning of the introduction on page 3 (see below):

*Two of these stressors that negatively affect mental health are noise and odour, e.g., the World Health Organization Regional Office for Europe (6) estimates that every year more than 1 million healthy years are lost due to traffic noise only. In addition, odour pollution is associated with many health complaints and decreased quality of life [7]. If we add to this the fact that mental disorders, and especially mood disorders, pose a very high burden on the population of Europe and are expected to become even more prominent for population health in the future [8, 9], it is clear that a better understanding of the relationship between odour and noise and mental health is needed in order to mitigate these noxious pathways.*

We ask the reviewer to point out where he/she feels that information about general health could be added.

(3) **on the missing values analysis (how was type of missingness determined?),**

In the new manuscript, we have added more information about the missing value analysis on page 11:

Using Little’s MCAR test, we found that the missing values in our analysis were not completely missing at random (as the p-value was significant at an alpha of 0.05).

To evaluate if there were differences in the missing values on psychological distress across the different sociodemographic groups, we used a cross tabulation to calculate the chi-square statistic. There were no significant differences in missing values among men and women, but those with lower levels of education and younger individuals were more likely to have missing values compared to those with higher educational levels and older individuals.

(4) **on reasons for not using imputation (in particular, regarding missing items in the K-10)**

In the previous letter, we explained why we chose listwise deletion over multiple imputation. We have now included the reasoning in the text on page 11:

We did not consider imputation methods for our missing values, because this is an estimation method which depends on the assumptions of the model that is estimated. Since we had a reasonable amount of missing values, we chose to rely on listwise deletion.

**A few other minor things that could still be ameliorated:**

**- mention that you did not have (for data protection reasons) the full addresses of the subjects (which would have allowed to model the environmental exposures, still a pity you couldn't)**

We have now mentioned this in the data and methods section on page 15:

Because we do not have access (for data protection reasons) to detailed address data, we cannot model actual noise and odour exposure for respondents. Alternatively, to get an indication of noise and odour exposure around the home, we modelled indicator variables for all districts and self-reported noise and odour annoyance around the home.

**- "noise exposure" rather than "objective level of noise"**

We used this terminology in order to make an explicit distinction between noise and odour exposure and the perceived noise and odour annoyance measures. However, we agree that noise exposure also captures this difference and have changed the terminology in the paper.

**- "stress reactions" rather than "stress"**

We agree that the term stress reaction is more accurate than stress and have changed the terminology in the paper.

**- get rid of the term "hearing issues", there are virtually no cases of hearing loss due to environmental noise (in contrast to industrial noise).**

This comment discusses the sentence below which was used to point out the health risks associated with exposure to noise and odour. We have now included concentration issues instead of hearing issues:

For the case of noise and odour, potential health risks are an increased probability of developing concentration or sleeping issues (for noise) or irritation on the eyes, nose and throat or respiratory issues (for odour) [27, 38, 39]. (page 6)

**- get rid of or change the sentence "Gender was measured with a dummy..:" This is nonsense: The variable "Gender" does not need to be transformed to a "dummy" as it only has two levels anyway! In statistical parlance, "dummies / dummy variables" are dichotomous variables (0 or 1) derived from a nominally scaled variable that has more than two levels. Usually for the sake of easier implementation in a statistical model and for a simpler interpretation of the coefficients. As an example, the nominal variable "sleep stage" with 6 different levels, namely W, S1, S2, S3, S4, REM can be broken down into 6 binary variables which can be used as predictors in a statistical model. If a person is e.g. in stage REM, that dummy variable gets the value REM=1, whereas all the others get the value 0. This is the reason behind "dummy". (I anyway prefer the term 'indicator variable' because it indicates the presense or absence of something).**

We understand the reviewers point and therefore changed the sentence into:

For gender, men formed the reference category. (page 14)

**- ad. "dummy" again: Check the sentence beginning with Following the study by van Deurzen... we used ... dummy variables... by using dummy variables"**

Conventions in our field are to call it dummy or dummy variable, but we understand that there are differences between scientific fields. We have therefore changed the terminology from dummy variable into indicator variables.
